# Rickettsiosis cases presenting with rash: a case series from an endemic region in Turkey

**DOI:** 10.1590/S1678-9946202567041

**Published:** 2025-06-27

**Authors:** Enes Dalmanoğlu, Mehmet Ali Tüz, Hande İdil Tüz, Derya Tuna Ecer

**Affiliations:** 1 Balıkesir University, Faculty of Medicine, Department of Infectious Diseases and Clinical Microbiology, Altıeylül, Balıkesir, Turkey; 2 Balıkesir Atatürk City Hospital, Department of Infectious Diseases and Clinical Microbiology, Altıeylül, Balıkesir, Turkey

**Keywords:** Rickettsia, Mediterranean spotted fever, Spotted fever, Tick-borne diseases

## Abstract

*Rickettsia* species are Gram-negative, pleomorphic coccobacilli that are obligate intracellular pathogens transmitted by arthropod vectors such as ticks. Among them, *Rickettsia conorii*, the causative agent of Mediterranean spotted fever (MSF), is endemic in many Mediterranean countries, including Turkey. This case series describes three patients from Balıkesir, Turkey, who developed high-grade fever, generalized maculopapular rash involving the palms and feet soles, arthralgia, and necrotic eschars (tache noire) at the tick bite sites. All cases occurred during summer and had documented exposure to *Rhipicephalus sanguineus*. Laboratory evaluations ruled out other tick-borne diseases, while real-time PCR performed on skin biopsy samples confirmed *Rickettsia* spp. Subsequent DNA sequencing of the gltA and ompA gene regions enabled species identification. Additionally, serological tests showed a significant rise in IgM and IgG antibody titers reacting with *Rickettsia conorii* antigen by indirect immunofluorescence assay. All patients were treated with doxycycline and recovered without complications. This case series highlights the importance of considering rickettsial infections in the differential diagnosis of febrile patients with rash and recent tick exposure, especially in endemic regions during warm seasons.

## INTRODUCTION

Rickettsiosis, an arthropod-borne infectious disease caused by *Rickettsia* species, is one of the oldest known zoonotic diseases affecting humans. Various arthropods, including lice, fleas, ticks, and mites, serve as transmission agents for *Rickettsia* species, the etiologic agents of rickettsial infections^1^. Mediterranean spotted fever (MSF), caused by *Rickettsia conorii* and transmitted by the brown dog tick (*Rhipicephalus sanguineus*), is a vector-borne disease characterized by fever, cutaneous eruptions, and the development of a black eschar (tache noire) at the tick bite site^2^. The tache noire lesion is widely regarded as a pathognomonic sign of MSF. The presence of this characteristic lesion, along with clinical manifestations and serological analysis, are the fundamental basis for MSF diagnosis^3^. Laboratory diagnosis of *Rickettsia* infections relies on serological, molecular, and culture-based methods. Among serological techniques, the indirect fluorescent antibody test (IFAT) and enzyme-linked immunosorbent assay (ELISA) are the most commonly used. IFAT is considered the gold standard for serological diagnosis; however, the presence of shared surface antigens among different *Rickettsia* species poses a challenge for species-specific differentiation at serological level^4^. Among molecular diagnostic methods, polymerase chain reaction (PCR), followed by DNA sequencing, is widely employed to identify and differentiate *Rickettsia* species. The most commonly targeted gene regions for species identification include the citrate synthase (gltA) gene and the genes encoding outer membrane proteins (ompA and ompB)^5,6^. This article presents three cases in which patients with a tick bite and rash history were evaluated by both molecular methods (PCR followed by DNA sequencing) and serological testing (IFAT), which revealed the presence of antibodies reacting with *Rickettsia conorii* antigen.

## CASE REPORTS

### Ethics

The patient's informed consent was obtained, and all procedures were conducted in accordance with the ethical principles of the Declaration of Helsinki.

### Case 1

**Figure 1:**
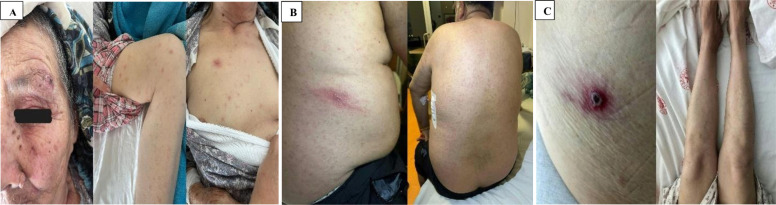
(A) In case 1, tache noire lesion on the left eyebrow and diffuse maculopapular rash on the body; (B) In case 2, tache noire lesion in the right lumbar region and diffuse maculopapular rash on the body; (C) In case 3, tache noire lesion on the anterior abdominal wall and diffuse maculopapular rash on the body.

A 79-year-old female patient presented to the emergency department of an external medical facility in June with a three-day history of periorbital inflammation and erythema. The patient also had a history of contact with a brown dog tick (*Rhipicephalus sanguineus*), which was observed on her left eyebrow and subsequently removed during examination. However, one week after the tick removal, the patient was readmitted to our hospital with fever, weakness, loss of appetite, a wound on the left eyebrow, joint pain, and a generalized rash. The patient exhibited a disseminated maculopapular rash involving the entire body, including palms and feet soles. The rash demonstrated blanching upon pressure and was non-pruritic (Figure 1A). The patient's general condition was moderate, with preserved consciousness, orientation, and cooperation. The body temperature was measured at 40 °C, and a tache noire-like lesion was observed over the left eye at the previous tick attachment site (Figure 1A). Serum and skin biopsy PCR for *Rickettsia* spp. were performed one week after disease onset. Indirect immunofluorescence assay was conducted both one week after disease onset and at the second week of treatment. Given the rash characteristics and the history of tick attachment, doxycycline 100 mg twice per day was initiated while awaiting further diagnostic evaluation for possible rickettsiosis. Following a 10-day course of doxycycline, no complications were observed, and the patient fully recovered without sequelae.

### Case 2

A 60-year-old male patient presented to the emergency department in June with complaints of fever, arthralgia, and rash. The patient's general condition was assessed as moderate, with preserved consciousness, orientation, and cooperation. His body temperature was recorded at 39.4 °C, while all other vital signs remained within normal limits. The patient had a history of contact with a brown dog tick (*Rhipicephalus sanguineus*), and reported having the tick removed from his back one week before admission. A necrotic wound was observed on the right side of the back at the tick removal site. Additionally, a widespread maculopapular rash was noted, extending to the palms and soles. The rash exhibited blanching upon pressure and was non-pruritic and non-painful (Figure 1B). Serum and skin biopsy PCR for *Rickettsia* spp. were performed four days after disease onset. Indirect immunofluorescence assay was conducted both four days after disease onset and at the second week of treatment. A course of doxycycline 100 mg twice per day was administered based on a presumptive rickettsiosis diagnosis, considering the history of tick bite and the presence of a tache noire-like lesion. The patient reacted positively to treatment, achieving full recovery without sequelae after a 10-day course of therapy.

### Case 3

A 50-year-old female patient presented to the emergency department in July with a history of tick removal 10 days prior, accompanied by fever, rash, malaise, and joint pain persisting for approximately one week. The patient's general condition was assessed as moderate, with preserved consciousness, orientation, and cooperation. Her body temperature was recorded at 39.2 °C. The patient had a history contact with a brown dog tick (*Rhipicephalus sanguineus*). Upon physical examination, a necrotic wound was observed on the anterior abdominal wall at the tick attachment site. Moreover, she had a diffuse maculopapular rash covering the entire body, including palms and feet soles. The rash was non-pruritic (Figure 1C). Serum and skin biopsy PCR for *Rickettsia* spp. were performed one week after disease onset. Indirect immunofluorescence assay was conducted both one week after disease onset and at the second week of treatment. Doxycycline 100 mg twice per day was initiated based on a presumptive rickettsiosis diagnosis, considering the documented history of tick bite and the presence of a tache noire-like lesion. The patient reacted positively to doxycycline treatment, fully recovering without complications or sequelae after a 10-day course of therapy.

### Laboratory tests

In all three cases, PCR results for Crimean-Congo Hemorrhagic Fever (CCHF), as well as *Borrelia* IgM and IgG tests, were negative. Additionally, no bacterial growth was observed in two sets of blood cultures obtained during the high fever period. The ticks removed from the patients were examined in the laboratory and identified as *Rhipicephalus sanguineus*. Table 1 presents the laboratory values of the patients at the time of admission to our hospital, along with the results of diagnostic tests for *Rickettsia* infection. Despite the negative results of the *Rickettsia* spp. real-time PCR test in serum samples, the skin biopsy yielded a positive result. DNA sequence analysis of the gltA and ompA gene regions was performed to identify *Rickettsia* species^7^. Biopsy samples from Patient 1's eyebrow lesion and eschar tissues from the other patients were used for this analysis. The DNA sequences obtained in this study were used for identification purposes only and were not of sufficient length or quality to warrant submission to public databases. Species identification was performed by comparing the sequence data to reference sequences using BLAST analysis.

**Table 1:** Biochemistry and hemogram results, *Rickettsia* real-time PCR, and indirect immune fluorescent antibody test results at the time of presentation.

Examination (unit) (normal range)		Case 1	Case 2	Case 3
Leukocyte count (×10^9^ / L) (4.5-11.0)		5,800	10,200	8,500
Platelet count (×10^9^ / L) (150,000-450,000)		**134,000**	187,000	392,000
Hemoglobin (g/dl) (12.0-16.0)		12.3	13.1	13.6
SGPT (U/L) (0-55)		17	43	**78**
SGOT (U/L) (5-34)		28	34	21
Total bilirubin (mg/dl) (0.2-1.2)		1.22	1.5	1.43
Lactic dehydrogenase (U/L) (125-243)		330	370	270
Creatine kinase (U/L) (30-200)		490	573	127
C-reactive protein (mg/L) (0-5)		68	92	51.6
Sedimentation (mm) (0-20)		69	71	65
Alkaline phosphatase (U/L) (40-150)		95	75	267
Prothrombin time (sec) (11-15)		12	17	15
INR (0.8-1.2)		1	1	1.18
Partial thromboplastin time (sec) (25-40)		35	33	39
*Rickettsia* spp. PCR				
	Serum	Negative	Negative	Negative
	Skin biopsy	Positive	Positive	Positive
*R. conorii* indirect immunofluorescence test: Initial serum sample				
	IgM	**1/192**	**1/192**	**1/192**
	IgG	**1/128**	**1/128**	**1/128**
*R. conorii* indirect immunofluorescence test: Serum sample after two weeks				
	IgM	**1/768**	**1/768**	**1/768**
	IgG	**1/1280**	**1/1280**	**1/1280**

SGOT = serum glutamate–oxaloacetate transaminase; SGPT = serum glutamate–pyruvate transaminase.

For the IFAT, a commercial kit (Vircell, Granada, Spain) coated with *Rickettsia conorii* antigen was used, and the procedure was performed according to the manufacturer's guidelines. The initial serum sample tested positive by the IFAT using *R. conorii* antigen, indicating the presence of antibodies reacting with *R. conorii* antigen. Due to known serologic cross-reactivity within the spotted fever group, species-level identification based on IFAT alone is limited. At the follow-up, conducted two weeks after treatment, the serum sample from the first patient demonstrated an IgM titer of 1/768 and an IgG titer of 1/1280, indicating a more than fourfold increase. To further confirm the serological diagnosis, Western Blot and Microimmunofluorescence Test (MIF) were performed. Results for the other cases are presented in Table 1. For DNA extraction, a polymerase chain reaction (PCR) was performed, followed by DNA sequence analysis. For sequence analysis, PCR amplification products were purified using the ExoSAP-IT™ PCR Product Cleanup Reagent (Thermo Fisher Scientific, ABD) according to the manufacturer's instructions. DNA sequencing was performed on an ABI 3730xl Sanger sequencing platform (Applied Biosystems, Foster City, CA, USA) using the BigDye Terminator v3.1 Cycle Sequencing Kit (Applied Biosystems, Foster City, CA, USA). The obtained DNA sequence data were analyzed using the Basic Local Alignment Search Tool (BLAST, version 2.0) to compare with reference sequences available in public databases. A punch biopsy specimen was homogenized using a Magnalyser homogenization device (Roche, Rotkreuz, Switzerland) with 500 μL of phosphate-buffered saline (PBS). A 100 μL portion of the homogenized tissue sample was then subjected to DNA extraction using a tissue extraction kit (Qiagen, Hilden, Germany). According to the established criteria for the interpretation of IFAT results, titers below 1/192 for IgM and 1/40 for IgG in a single serum sample were considered negative^8^. In accordance with the ESCMID *Coxiella*, *Anaplasma*, *Rickettsia*, and *Bartonella* (ESCAR) Working Group's diagnostic guidelines for tick-borne bacterial infections, a total score exceeding 25 is required for the diagnosis of MSF caused by *Rickettsia conorii.* In this study, the total score obtained for each case surpassed this threshold, thereby supporting the ESCAR Working Group's diagnostic criteria^9^ (Table 2).

**Table 2:** Patients’ score according to the ESCAR study group criteria for the diagnosis of Mediterranean spotted fever^9^.

Criteria		Score^a^		
Epidemiological criteria		Case 1	Case 2	Case 3
	Residence in an endemic area	2	2	2
	Occurrence during May–October	2	2	2
	Contact (certain or possible) with dog ticks	2	2	2
**Clinical criteria**				
	Fever >39 °C	5	5	5
	Eschar (Tache noire)	5	5	5
	Maculopapular or purpuric rash	5	5	5
	Two of the above criteria	0	0	0
	All three of the above criteria	5	5	5
**Non-specific laboratory findings**				
	Platelet count <150,000/mm^3^	1	0	0
	SGOT or SGPT >50 U/L	0	0	1
**Bacteriological criteria**				
	Positive blood culture for Rickettsia conorii	0	0	0
	Rickettsia conorii detection in a skin biopsy	0	0	0
**Serological criteria**				
	Single serum and IgG >1/128	0	0	0
	Single serum and IgG >1/128 and IgM >1/64	10	10	10
	Four-fold increase in two sera obtained within a two-week interval	20	20	20
**Total Points**		57	56	57

SGOT = serum glutamate–oxaloacetate transaminase; SGPT = serum glutamate–pyruvate transaminase;

a A positive diagnosis is made when the overall score is ≥25.

## DISCUSSION

Mediterranean spotted fever is a tick-borne disease caused by *Rickettsia conorii*, which is widely distributed throughout Mediterranean countries. The disease is characterized by fever, a maculopapular rash, and the presence of a tache noire at the tick bite site^10^. Elevated body temperature is commonly accompanied by the onset of a rash that typically begins on extremities, including palms and feet soles. Concurrently, a maculopapular rash may develop, with the potential for petechial lesions^11^. The cases presented with maculopapular rashes and exhibited several shared characteristics, including occurrence between May and October, residence in an endemic region, history of tick exposure, and involvement in animal husbandry and farming activities^12^. The entire cohort was admitted to the hospital during summer. The MSF diagnosis is typically based on an algorithmic approach that integrates epidemiological, clinical, and laboratory criteria. In Europe, the ESCAR Working Group has established diagnostic criteria for MSF, specifying that a total score above 25 indicates an increased risk of tick-borne diseases. In accordance with these criteria, the total score for each case in this study exceeded 25 points, thereby indicating a high probability of tick-borne disease. Earlier studies conducted in Thrace reported that maculopapular rashes commenced within 12 h to 10 days after fever onset. Rash on the palms and feet soles was identified in 84.4% of cases, while eschar was found in 70.3%^13^. In all cases, the rash appeared following fever onset. Concurrently, tache noire lesions and maculopapular rashes were observed on the palms and soles. In a study including 56 patients with MSF, Şengöz *et al*.^12^ reported thrombocytopenia in 25% of cases and abnormal liver function tests in a higher percentage of patients. One patient exhibited thrombocytopenia, while another demonstrated elevated liver function test results. Although an MSF diagnosis can be made based on the patient's history and physical examination findings, additional laboratory tests may be required to confirm rickettsiosis. These tests include serological, molecular, and culture-based methods. Rickettsiosis diagnosis can be facilitated by various techniques, including IFA, latex agglutination, ELISA, indirect immunoperoxidase, and Western blot. However, IFAT is widely regarded as the gold standard for rickettsiosis serological diagnosis^8^. IgM-type antibodies typically emerge early in the course of infection and gradually decline over several weeks. Conversely, IgG-type antibodies begin rising from the second week of illness and, in some cases, may persist at low levels for an extended period, often spanning years^14^. Serum samples obtained from all three patients tested positive for antibodies reacting with *Rickettsia conorii* antigen at the appropriate titers by IFAT. Molecular diagnostic methods can be performed on whole blood and buffy coat samples; however, it is important to note the PCR assay has demonstrated reduced sensitivity in blood samples, likely due to transient bacteremia. In contrast, tissue biopsy samples have shown to yield more reliable positive results. Therefore, using punch biopsy specimens from the tache noire lesion or swab specimens from eschar exudate holds significant diagnostic value in confirming *Rickettsia* infections^15^. Real-time PCR analysis of skin biopsy samples confirmed the presence of *Rickettsia* spp. in all cases. The indirect immunofluorescence assay, employed for diagnostic purposes, enables differentiation between typhus group and spotted fever group rickettsiae. However, species-level distinction remains challenging due to significant cross-reactivity within the spotted fever group. Both *Rickettsia rickettsii* and *Rickettsia conorii*, belonging to the spotted fever group, exhibit antigenic similarity and cross-reactivity, making differentiation by IFAT difficult. However, their distinct epidemiological distribution provides a useful diagnostic clue. *R. rickettsii* is predominantly found in the Americas, whereas *R. conorii* is primarily identified in the Mediterranean^13,16,17^. In accordance with the aforementioned findings, the *Rickettsia* species detected in infections within our country has been identified as *Rickettsia conorii*. Although *R. conorii* is considered the predominant Spotted Fever Group (SFG) *Rickettsia* species in the Mediterranean, other members of this group, such as *R. massiliae*, *R. monacensis*, and *R. slovaca* have also been associated with similar clinical conditions, including fever, rash, and eschar formation. The serological cross-reactivity between these species further complicates species-level diagnosis using IFAT alone. Therefore, molecular confirmation by gene sequencing remains crucial, particularly in regions where multiple SFG *Rickettsia* species may coexist^18^. Based on the methodology employed in our case series, doxycycline is considered the primary antibiotic treatment option for MSF. A clinical response to treatment is typically observed by the second or third day after initiation. The treatment duration generally ranges between seven and 10 days^19^.

## CONCLUSION

MSF may be a possible diagnosis in patients presenting with fever, rash, headache, malaise, maculopapular rash, and myalgia, particularly in endemic regions and during seasons when tick bites are common. It is essential to recognize that our country is an endemic region for MSF and, therefore, MSF should be included in the differential diagnosis of patients exhibiting symptoms consistent with the disease. However, confirmation by specific diagnostic methods is crucial for accurate diagnosis and appropriate management.
